# A 21-Year-Old Student with Fever and Profound Jaundice

**DOI:** 10.1371/journal.pntd.0002534

**Published:** 2014-01-09

**Authors:** Samson Ejiji Isa, Kenneth Ikenna Onyedibe, Mark Ojogba Okolo, Abiayi Elmina Abiba, Johnson Simon Mafuka, Gomerep Samuel Simji, Shehu Yakubu Nathan, Ubong Aniefok Udoh, Sati Klein Awang, Daniel Zanyu Egah, Edmond Banle Banwat, Melanie Newport, Ahmed Ahmed

**Affiliations:** 1 Infectious Diseases Unit, Department of Medicine, Jos University Teaching Hospital, Jos, Plateau State, Nigeria; 2 Faculty of Medical Sciences, University of Jos, Jos, Plateau State, Nigeria; 3 Department of Medical Microbiology, Jos University Teaching Hospital, Jos, Plateau State, Nigeria; 4 Leptospirosis Unit, Central Diagnostics Laboratories National Veterinary Research Institute, Vom, Plateau State, Nigeria; 5 Department of Infectious Diseases, Brighton and Sussex University Hospital, Brighton, Southeast England, United Kingdom; 6 Global Health Programme, Brighton and Sussex University, Brighton, Southeast England, United Kingdom; 7 Royal Tropical Institute, KIT Biomedical Research, WHO/FAO/OIE and National Collaborating Center for Reference and research on Leptospirosis, Meibergdreef, Amsterdam, The Netherlands; Universidad Peruana Cayetano Heredia, Peru

## Description of Case

A 21-year-old male student who resides in a university hostel in North Central Nigeria presented to our emergency unit with one-week history of fever, upper abdomen pain for five days, and yellow discoloration of the eyes for two days. Fever was high grade, intermittent, and associated with chills and rigors, anorexia, malaise, muscle and joint pain, sore throat, and a throbbing headache. He had no history of previous hospital admissions, surgery, blood transfusion, or sickle cell disease. There was no history of travels, direct contact with animals, ingestion of unhygienic water or food, or history of unprotected sex. He resided in a hostel where there was recent flooding, which was also infested with rodents and had livestock roaming freely. He denied knowledge of similar illness among his close contacts and those in his vicinity. He did not smoke cigarettes, drink alcohol, or use recreational drugs and was uncertain about previous vaccinations but had always been in good health. He was referred from a primary care hospital on account of the above symptoms after initial resuscitation and investigations.

On examination, he appeared well nourished but acutely ill, deeply icteric, febrile (39.1°C), with normal oropharynx, and without palpable lymphadenopathy or rash. The only positive abdominal finding was a palpable tender liver 6 cm below the right costal margin with a span of about 15 cm. There was no localized tenderness, and Murphy's sign was negative. Other systems were normal, except for tachycardia of 128 beats per minute.

His initial liver biochemistry was as follows: total bilirubin, 113.9 µmol/L (3.4–17 µmol/L); conjugated bilirubin, 86.7 µmol/L (1–8 µmol/L); alkaline phosphatase, 146 IU/L (21–92 IU/L); alanine transaminase (ALT), 109 IU/L (1–40 IU/L); aspartate transaminase (AST), 88 IU/L (1–40 IU/L). The electrolytes were sodium 107 mmol/L (134–145 mmol/L), potassium 3.5 mmol/L (3.5–5.5 mmol/L), creatinine 208 µmol/L (72–126 µmol/L), and urea 3.5 mmol/L (2.5–6.6 mmol/L). Complete blood count revealed a total white cell count (WCC) of 12.0×10^3^/µL (2.5–11×10^3^/µL) with a neutrophilia of 90% and platelets of 309×10^3^/µL (90–400×10^3^/µL). The abdominal ultrasound scan done three days after admission was normal. The random blood sugar and clotting profile were within normal limits, while HIV screen was negative, and urine culture result was pending.

## What Are Three Additional Laboratory Tests You Would Request to Make a Diagnosis?

1. Urinalysis
2. Blood cultures
3. Malaria parasite microscopy

## What Are the Important Differential Diagnoses?

Differential diagnosis of fever with jaundice is broad but knowledge of local disease epidemiology can point to the relevant differentials. A short history of fever, upper abdominal pain, and profound jaundice which was preceded by flu-like symptoms could suggest initial features of viral haemorrhagic fevers (VHFs) in sub-Saharan Africa where the risks for disease outbreak are always present [1].The common VHFs in Nigeria are yellow fever (YF) and Lassa fever (LF). Although the last YF epidemic in Nigeria was in 1995, the less alarming but important endemic form of YF which may precipitate an outbreak when herd immunity is low could be occurring unnoticed. Whereas this patient has markedly elevated conjugated bilirubin with only modestly elevated alkaline phosphatase, as seen in YF, the absence of relative bradycardia, leucopenia, thrombocytopenia, coagulopathy, azotaemia, and albuminuria all suggest alternative diagnosis. Indeed, albuminuria is an important discriminator between YF and other endemic causes of acute viral hepatitis [2]. The IgM-ELISA is the most widely used serologic test for diagnosis where a single positive test is presumptive of YF and a 4-fold rise in paired serum samples is confirmatory of YF [1]. Unfortunately, this test is not readily available in Nigeria, and where available, the costs are prohibitive. LF was first recognized in Nigeria in 1969, and there have been frequent outbreaks since then. This patient was exposed to rats, the vectors for LF, and presented with high grade fever associated with prodromal flu-like symptoms and had normal platelet counts as usually seen in LF compared with other VHFs. However, the deep jaundice, together with markedly elevated bilirubin and the absence of retrosternal pain, exudative pharyngitis as objective evidence of sore throat, albuminuria, and coagulopathy, makes LF unlikely [3].

Community-acquired sepsis (CAS) is an important differential diagnosis in a previously healthy young man presenting with fever and jaundice in the tropics. A large study in the HIV/AIDS era showed a CAS prevalence of 16% among febrile new admissions, where non-typhi salmonellae (NTS) and *Streptococcus pneumonia* predominated [4]. Before the HIV/AIDS epidemic, *Salmonella typhi* greatly outnumbered NTS in adults, and enteric gram-negative organisms formed a greater proportion of total gram-negative isolates [5]

Enteric fever (EF), caused by *S. typhi* and *S. paratyphi*, is a gram-negative septicaemic illness characterized by fever, headache, anorexia, myalgias, and intestinal symptoms and signs. It is endemic in developing countries and is transmitted faeco-orally through consumption of contaminated food or water or direct person-to-person contact and is more likely than NTS to cause severe illness in immunocompetent hosts. However, there was no known history of exposure, diarrhoea, or constipation in this patient, and his WCC was raised as compared to normal or reduced counts seen in EF. While ALT and AST are raised 2–3 times above upper limit of normal, bilirubin levels are normal or only slightly raised in EF. Importantly, the cultures of blood, urine, and stools did not yield *Salmonella* species.

Severe malaria was also in consideration because the disease is endemic in Nigeria, and commonly presents with fever, headaches, aches, and chills and rigors in adults. However, severe malaria, which is almost exclusively caused by *Plasmodium falciparum*, is usually seen in pregnant women, young African children under the age of 5 years, and in non-immune travellers who acquire infection in endemic countries [6]. In susceptible individuals, massive intravascular haemolysis can result in release of free haemoglobin which may lead to acute renal failure. Severe malaria is an unlikely diagnosis in this adult, semi-immune patient. In addition, the markedly elevated conjugated serum bilirubin with normal haemoglobin and platelets and negative thick and thin blood films for malaria parasites all suggest an alternative diagnosis [7], [8].

Amoebic liver abscess is a tropical parasitic disease which is much more common in male adults. It typically presents with high grade fever that may be associated with rigors and profuse sweating, upper abdominal pain, tender hepatomegaly, leucocytosis, and raised alkaline phosphatase. Pulmonary and pericardial features may also be seen if abscess ruptures into the thoracic cavity, and may, less commonly, rupture into other abdominal viscera, inferior vena cava, and the skin. Whereas concurrent rectocolitis is unusual, hepatic abscess is likely the result of metastasis of *Entamoeba histolytica* trophozoites from the colon via the portal vein to the liver [9]. Jaundice is seen in less than a quarter of cases and when present, indicates an abscess sufficiently large enough to obstruct the hepatobiliary tree. Ultrasound is an excellent investigation that demonstrates filling defect and is therefore unlikely to miss large abscesses that can be expected in those with jaundice.

Acute cholecystitis or choledocholithiasis with ascending cholangitis were also considered because of the short onset of fever associated with chills and rigors, upper abdominal pain, jaundice, neutrophilia, and a largely cholestatic liver biochemistry. However, Murphy's sign was negative, and abdominal scan showed normal hepatobiliary tree. In addition, absence of alcohol abuse and features of other risk factors like suppurative appendicitis effectively ruled out pyogenic liver abscess.

Nigeria is hyperendemic for acute hepatitis A to E viruses, and apart from hepatitis E virus whose antibody prevalence is unexpectedly highest in young adults, most cases occur in children, who are mostly asymptomatic, and lifelong immunity ensues as with hepatitis A infection. Hepatitis E outbreak may follow flooding, but our patient's hepatitis E IgM antibody may have been falsely positive [10], as substantial improvement of prodromal symptoms with appearance of jaundice, leucopenia instead of neutrophil leucocytosis, and markedly elevated liver transaminases in the range of 1000–2000 IU/L are seen in acute viral hepatitis [10].

A combination of fever, headache, myalgia, and hepatic dysfunction is also seen in rickettsial and Q fevers, as well as in brucellosis. Nigeria is not known to be endemic for most rickettsioses, and our patient lacked the appropriate exposures for these diseases.

Leptospirosis was considered a likely diagnosis based on the history of exposure to floodwaters contaminated by livestock and rats, and a prodromal flu-like syndrome associated with appearance of deep jaundice while still febrile. In leptospirosis, there is a disproportionate increase in conjugated bilirubin accompanied by modest elevations in transaminases which rarely exceed 200 IU/L because hepatic necrosis is uncommon [11].

## What Are the Clinical Syndromes of Leptospirosis?

The incubation period is between 4–20 days, and infection is associated with a broad spectrum of severity, ranging from subclinical illness to two clinically recognizable syndromes—a self-limited systemic illness seen in approximately 90% of infections and a severe, potentially fatal illness accompanied by any combination of renal failure, liver failure, and pneumonitis with hemorrhagic diathesis [12], [13]. The virulence of the infecting leptospire and the host immune mechanisms appear to be important factors in the severity of illness.

Leptospirosis is classically described as a biphasic illness comprising of an initial septicemic stage followed by a temporary decline in fever and an immune phase in which the severe symptoms occur. However, in many severe cases, the distinction between these two phases is not apparent; in addition, many patients present only with the onset of the second phase of the illness [14]. The acute septicemic phase of illness begins abruptly with a high remittent fever (38°–40°C) and other non-specific symptoms and lasts for 5–7 days. The immune phase of illness generally lasts from 4–30 days and coincides with the appearance of IgM antibodies [13]. The most distinctive syndrome of severe illness that may develop after the acute phase of illness is Weil's disease, characterized by impaired hepatic and renal function. Other severe manifestations include hyperpyrexia, hemorrhagic pneumonitis, cardiac arrhythmia, or circulatory collapse [13].

In addition to the hepatocellular pattern of hepatic dysfunction with disproportionately elevated bilirubin as compared to serum transaminases seen in our patient, there was impaired renal function with creatinine at 208 µmol/L, sodium 107 mmol/L, and potassium 3.5 µmol/L at the lower limit of normal. Initial kidney involvement in leptospirosis is characterized by a unique non-oliguric hypokalaemic renal impairment whose hallmarks are impaired sodium reabsorption and potassium wasting [14].

## How Would You Make a Diagnosis of Leptospirosis?

Leptospirosis should be suspected on the basis of an appropriate exposure history combined with any of the infection's protean manifestations.

Direct visualization of leptospires in blood or urine by darkfield microscopic examination has been used for diagnosis. However, artefacts are commonly mistaken for leptospires, and the method has both low sensitivity (40.2%) and specificity (61.5%) [15].

Leptospires can be isolated from blood, cerebrospinal fluid (CSF), and peritoneal dialysate fluids during the first ten days of illness. Normally, specimens should be collected while the patient is febrile and before antibiotic therapy is initiated. Urine can also be cultured after the first week of illness. Cultures are performed in albumin-polysorbate media such as Ellinghausen-McCullough-Johnson-Harris (EMJH) medium. Cultures are incubated at 30°C for several weeks because initial growth may be very slow. Isolated leptospires are identified to serovar level by traditional serologic methods or by molecular methods [14].

Most cases of leptospirosis are diagnosed by serology. The reference standard assay is the microscopic agglutination test (MAT), in which live antigens representing different serogroups of leptospires are reacted with serum samples and then examined by darkfield microscopy for agglutination [16]. A serologically confirmed case of leptospirosis is defined by a 4-fold rise in MAT titer to one or more serovars between acute-phase and convalescent serum specimens run in parallel. However, a single titer of at least 1∶200 obtained after the onset of symptoms also suggests recent or current infection with leptospirosis [17]. The MAT is a serogroup-specific assay and should not be used to infer the identity of the infecting serovar [18], but knowledge of the presumptive serogroup may be of epidemiologic value in determining potential exposures to animal reservoirs.

Another serologic method for diagnosis of leptospirosis is an assay for IgM antibodies, which are detectable after about the fifth day of illness [19], [20]. Positive IgM assays are not confirmatory for leptospirosis but serve as screening tests. These antibody assays have the potential to enhance the diagnostic capacity of many laboratories, particularly in developing countries, where most cases occur [14].

**Figure 1:**
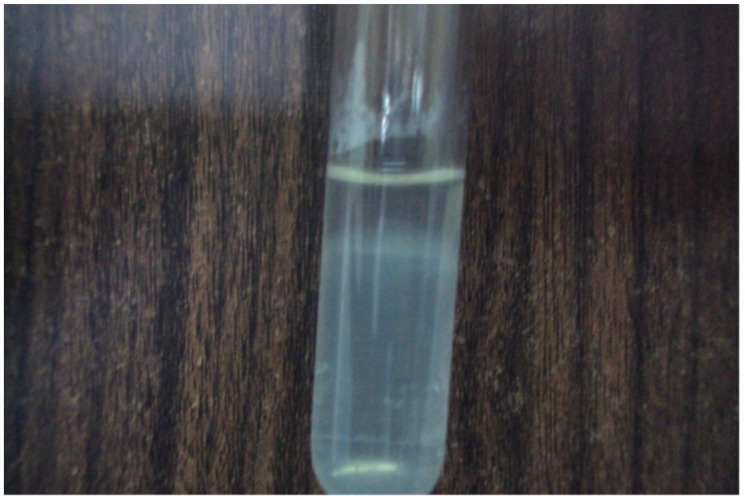
Dinger's ring (the opaque ring just below the surface of the semi-solid EMJH media in the tube) seen on the ninth day of culture.

**Figure 2:**
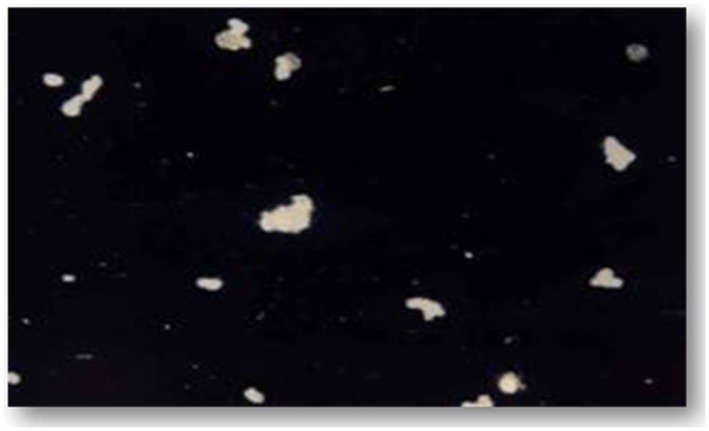
Positive microscopic agglutination test (MAT) at a titre of 1∶640.

**Figure 3:**
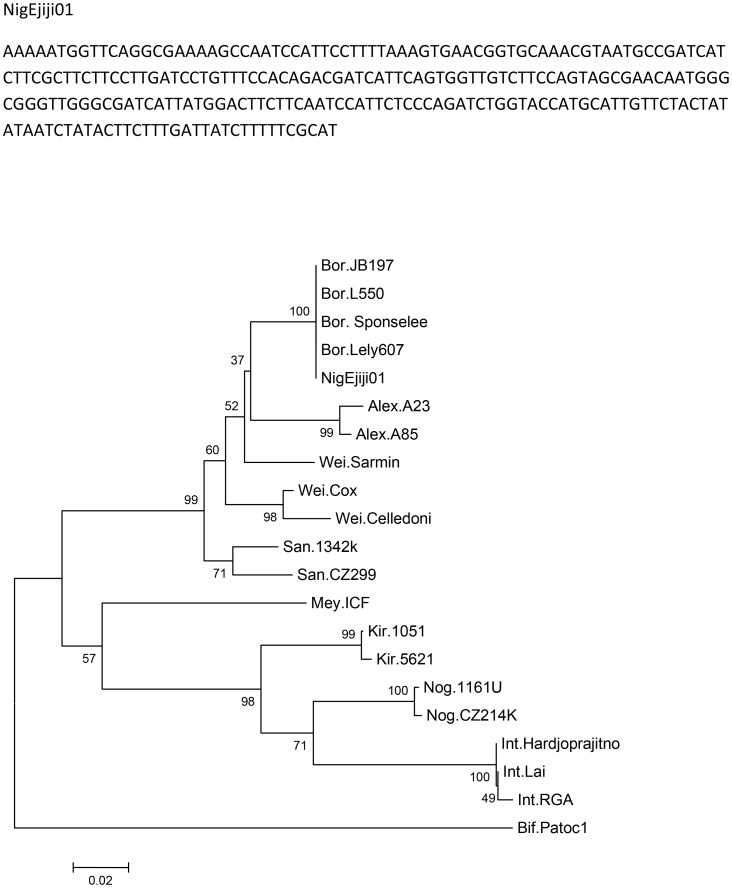
Genetic sequence and evolutionary relationships of the isolate (NigEjiji01) in the *Leptospira* taxa. Bor. = *L. borgpetersenii*, Alex. = *L. alexanderi*, Wei. = *L. weilii*, San. = *L. santarosai*, Mey. = *L. meyeri*, Kir. = *L. kirschneri*, Nog. = *L. noguchii*, Int. = *L. interrogans*, Bif. = *L. biflexa*.

The patient had urine and blood samples sent to a reference laboratory. Culture of the urine sample on EMJH medium showed the characteristic Dinger's ring by the ninth day (Figure 1). MAT was performed by standard methods [21] using a panel containing *Leptospira* serovars Icterohaemorrhagiae, Tarassovi, Pomona, Grippotyphosa, Bratislava, Canicola, Hardjo, Projitno, and Mini. The reaction was observed with darkfield microscopy and the results were positive for *L. borgpetersenii* antibody at titre of 1∶640 (Figure 2). Serotyping of the isolate was carried out by standard methods [16] and results were positive for *L. borgpetersenii* serovar Tarassovi. Specie confirmation by phylogenetic partial *secY* sequences (Figure 3) was carried out at the Leptospirosis Reference Centre, Amsterdam according to Victoria et al. [22]. The evolutionary history was inferred using the Neighbor-Joining method [23]. The optimal tree with the sum of branch length = 0.76510704 is shown. The percentages of replicate trees in which the associated taxa clustered together in the bootstrap test (2000 replicates) are shown next to the branches [24]. The evolutionary distances were computed using the Maximum Composite Likelihood method [25] and are in the units of the number of base substitutions per site. The analysis involved 21 nucleotide sequences. Evolutionary analyses were conducted in MEGA5 [26].

## How Would You Treat This Patient?

Antibiotic therapy should be initiated as early in the course of the disease as suspicion allows. Its prompt initiation probably shortens the course of severe leptospirosis and prevents the progression of mild disease [14]. Antimicrobial drugs (typically penicillin, ceftriaxone, or cefotaxime) should be used to treat severe later-stage leptospirosis, and mild disease should be treated with oral doxycycline (Table 1). Jarisch-Herxheimer reactions have been reported in patients treated with penicillin [27], and such patients should be monitored closely because of the increased morbidity and mortality from such reactions. Supportive therapy is essential for hospitalized patients, especially those with renal dysfunction and severe pulmonary haemorrhage syndrome.

**Table 1:** [14] Treatment of leptospirosis in adults.^a^

Indication	Regimen
Mild leptospirosis	Doxycycline (100 mg PO bid) *or*
	Amoxicillin (500 mg PO tid) *or*
	Ampicillin (500 mg PO tid)
Moderate/severe leptospirosis	Penicillin (1.5 million units IV or IM q6h) *or*
	Ceftriaxone (1 g/d IV) *or*
	Cefotaxime (1 g IV q6h)

^a^ All regimens are given for seven days.

The patient was commenced on intravenous ceftriaxone right from admission. He recovered fully and was discharged on the eighth day.

## Discussion

Leptospirosis is a zoonosis of global distribution caused by infection with pathogenic spirochetes of the genus *Leptospira*. The disease is particularly underreported in tropical regions where it is endemic and is often mistaken for other febrile illnesses. Although serovars continue to adapt to new hosts [28], the domestic mouse *Mus musculus* is a reservoir for *L. borgpetersenii* of the serovar Ballum [29], [30] and pigs are reservoirs for the serovar Tarassovi [31]. Human infection occurs by direct contact with infected animal urine or tissues or, more commonly, by indirect exposure to the organisms in contaminated damp soil or water. Our patient may have been infected through direct exposure to urine of rodents in his hostel or through exposure to floodwaters contaminated by animals, including pigs.

Whereas leptospirosis outbreaks are to be expected in situations where large numbers of persons are exposed to contaminated water [32], differences in virulence of leptospire strains, individual immune response variation to infection, lack of disease awareness on the part of health providers, and weak referral and notification systems might have contributed to failure to recognise other possible cases. A high index of suspicion is necessary in diagnosing leptospirosis, and suspected cases should be commenced on antibiotics as its prompt initiation shortens the course of severe disease and prevents progression to severe illness. To our knowledge, this is the first case of human leptospirosis confirmed at species level in Nigeria and the first reported case implicating the serovar Tarassovi.

### Consent

We obtained an informed written consent from the patient to have his case published.

### Accession number

The GenBank accession number for our nucleotide sequence is BankIt1627333 Leptospira KF039884.

**Learning Points**

- Leptospirosis is an important differential diagnosis of acute febrile illnesses in developing countries like Nigeria.
- Leptospirosis mimics many common febrile tropical illnesses like viral hepatitis, severe malaria, and enteric fever.
- The clinical manifestations of leptospirosis are protean, and a high index of suspicion is needed to make a diagnosis and commence appropriate therapy.
- Leptospirosis is potentially fatal but easy to treat if diagnosed early.
- At the population level, it is important to improve general hygiene standards to reduce contact with the organism.
